# Activation of surrogate death receptor signaling triggers peroxynitrite-dependent execution of cisplatin-resistant cancer cells

**DOI:** 10.1038/cddis.2015.299

**Published:** 2015-10-22

**Authors:** S Seah, I C C Low, J L Hirpara, K Sachaphibulkij, G Kroemer, C Brenner, S Pervaiz

**Affiliations:** 1 Department of Physiology, Yong Loo Lin School of Medicine, National University of Singapore, Singapore; 2 NUS Graduate School for Integrative Sciences and Engineering, National University of Singapore, Singapore; 3 Cancer Science Institute, National University Health System, Singapore; 4 Equipe 11 Labellisée par la Ligue Contre le Cancer, Centre de Recherche des Cordeliers, Paris, France; 5 Cell Biology and Metabolomics Platforms, Gustave Roussy Comprehensive Cancer Center, Villejuif, France; 6 INSERM, U1138, Paris, France; 7 Université Paris Descartes, Sorbonne Paris Cité, Paris, France; 8 Université Pierre et Marie Curie, Paris, France; 9 Pôle de Biologie, Hôpital Européen Georges Pompidou, AP-HP, Paris, France; 10 INSERM UMR-S 1180, LaBex LERMIT, University of Paris Sud, Châtenay-Malabry, France; 11 National University Cancer Institute, National University Health System, Singapore; 12 School of Biomedical Sciences, Curtin University, Perth, Western Australia, Australia

## Abstract

Platinum-based drugs remain as the cornerstone of cancer chemotherapy; however, development of multidrug resistance presents a therapeutic challenge. This study aims at understanding the molecular mechanisms underlying resistance to cisplatin and unraveling surrogate signaling networks that could revert sensitivity to apoptosis stimuli. We made use of three different sets of cell lines, A549 and H2030 non-small-cell lung cancer (NSCLC) and A2780 ovarian cancer cells and their cisplatin-resistant variants. Here we report that cisplatin-resistant cell lines displayed a multidrug-resistant phenotype. Changes in mitochondrial metabolism and defective mitochondrial signaling were unraveled in the resistant cells. More interestingly, a marked increase in sensitivity of the resistant cells to death receptor-induced apoptosis, in particular TRAIL (TNF-related apoptosis-inducing ligand)-mediated execution, was observed. Although this was not associated with an increase in gene transcription, a significant increase in the localization of TRAIL death receptor, DR4, to the lipid raft subdomains of plasma membrane was detected in the resistant variants. Furthermore, exposure of cisplatin-resistant cells to TRAIL resulted in upregulation of inducible nitric oxide synthase (iNOS) and increase in nitric oxide (NO) production that triggered the generation of peroxynitrite (ONOO^−^). Scavenging ONOO^−^ rescued cells from TRAIL-induced apoptosis, thereby suggesting a critical role of ONOO^−^ in TRAIL-induced execution of cisplatin-resistant cells. Notably, preincubation of cells with TRAIL restored sensitivity of resistant cells to cisplatin. These data provide compelling evidence for employing strategies to trigger death receptor signaling as a second-line treatment for cisplatin-resistant cancers.

Platinum-based chemotherapeutics belong to a class of alkylating agents widely used in the treatment of a variety of human malignancies such as lung, ovarian, testicular, bladder, head and neck and other sarcoma-derived cancers.^1^ The first such agent, cisplatin, was initially discovered for its ability to inhibit DNA synthesis and cause filamentous growth in *E. coli*,^2^ a common property of platinum-based compounds that confers them with antiproliferative potential. Despite its potent antiproliferative property, the clinical use of cisplatin is hampered by its high toxicity profile.^3, 4, 5^ Therefore, newer generation of cisplatin analogs with lower toxicity such as carboplatin are currently the preferred choice.

Platinum-based drugs form reactive intermediates that form DNA intra-strand crosslink adducts^1, 6^ that in turn activates DNA repair response. However, platinum-DNA adducts are usually poorly repaired,^7^ often resulting in the activation of DNA damage response and cell death. The best characterized cell death pathway triggered by cisplatin-induced DNA damage involves p53-dependent transcription of pro-apoptotic Bcl-2 family members (Puma, Noxa, Bax) that trigger death execution via the mitochondria apoptotic pathway.^8, 9, 10^

Although platinum-based therapy may be commonly prescribed in anticancer regimens, its efficacy is limited by the high incidence of chemoresistance. More worryingly, cross-resistance to other chemotherapeutic agents limits chemotherapy options against refractory tumors. Efforts into understaning the underlying mechanism(s) of cisplatin resistance in cancer cells have unraveled several mechanisms including but not limited to: (1) enhanced DNA repair machinery that allows cancer cell to evade genotoxic stress,^11^ (2) increase in extrusion of platinum drugs via the expression of plasma membrane transporters^12, 13, 14^ and (3) loss of p53 function and alterations in Bcl-2 family protein profiles that allow cancer cells to evade apoptosis.^15, 16^

Despite the increased understanding of how tumor cells acquire resistance to platinum-based compounds, there are still limited second-line treatment options/strategies for patients who relapse and/or are refractory to conventional chemotherapeutics. Here we report that non-small-cell-lung carcinoma (NSCLC) and ovarian cancer cells rendered resistant to cisplatin harbor a rewired oncogenic network that allows the cells to evade mitochondria apoptotic execution on one hand but sensitizes the malignant cells to apoptotic signaling via the death receptors on the other. The augmented sensitivity of cisplatin-resistant cells to TRAIL (TNF-related apoptosis-inducing ligand) signaling results from the redistribution of the death receptor 4 (DR4) to lipid raft subdomains within the plasma membrane. More importantly, pretreatment of cisplatin-resistant cancer cells with the death receptor agonist, TRAIL, restores sensitivity of refractory cancer cells to cisplatin. Our work highlights the potential of death receptor agonists, with TRAIL in particular, as a potential second-line chemotherapy for the treatment of recurrent/refractory malignancies.

## Results

### Cisplatin-induced apoptosis is blunted in R1 and R2 cells

**Figure 1:**
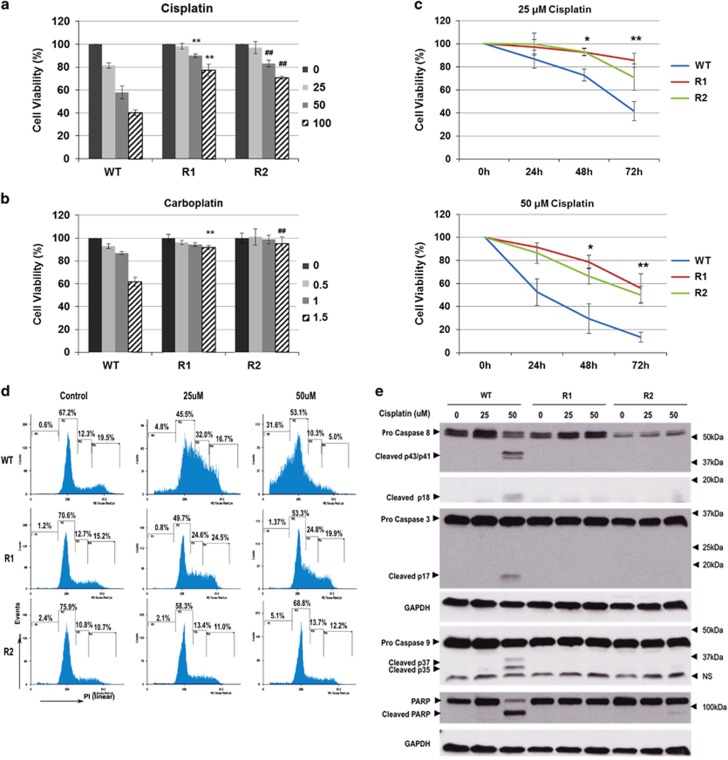
Sensitivity of A549 WT, R1 and R2 cells to platinum-based drugs. Cells were treated with (**a**) cisplatin (*μ*M) or (**b**) carboplatin (mM) at the indicated doses for 24 h and cell viability was assessed by MTT assay. *P*<0.01 compared with WT treated at respective dose. (**c**) WT, R1 and R2 cells were treated with various concentrations of cisplatin for 24, 48 and 72 h. Cell viability was determined by MTT assay and expressed as % of untreated control. (**d**) Cells were treated with increasing doses of cisplatin for 24 h and DNA content was analyzed by flow cytometry after staining with propidium iodide (PI); y axis: events, x axis: PI linear fluorescence, *n*=3. (**e**) WT, R1 and R2 cells were treated with the indicated doses of cisplatin for 24 h and caspase 3, 8 or 9 processing and PARP cleavage was determined by western blotting. Data shown are mean±S.D. of at least three independent experiments. In panels **a** and **b**: ** and ^##^ indicate *P*-value<0.005 compared to cisplatin sensitivity of WT cells. In panel **c**: * and ** indicate *P*-value<0.05 compared to WT cells treated with cisplatin for 48 and 72h, respectively

Two cisplatin-resistant cell-lines, R1 and R2, were generated via single clone selection of A549 NSCLC parental cell line by continuous exposure to low-dose (5 *μ*M) cisplatin for 6 months. Wild-type A549 (WT) cells exhibited a cohesive and clustered appearance, whereas the resistant clones (R1 and R2 cells) grew in a sparse and disorderly manner (Supplementary Figure S1A). Compared with the WT cells, R1 and R2 clones were relatively resistant to cisplatin (Figures 1a and c) and exhibited cross-resistance to another platinum-based compound, carboplatin (Figure 1b). In line with this, propidium iodide (PI) staining of cisplatin-treated cells revealed higher DNA fragmentation, as indicated by the increased sub-G1 population, in cisplatin-treated WT cells relative to R1 and R2 cells (Figure 1d). In addition, caspases 8, 9 and 3 were activated in cisplatin-treated WT cells but not in the cisplatin-resistant clones. PARP (poly(ADP-ribose) polymerase), a substrate of caspase 3, was also cleaved in WT but not R1 and R2 cells (Figure 1e). Finally, cisplatin-induced inhibition of long-term colony formation in WT cells was significantly abrogated in R1 and R2 cells (Supplementary Figure S1D).

### Cisplatin-resistant cells exhibit cross-resistance to triggers that engage the intrinsic death pathway

**Figure 2:**
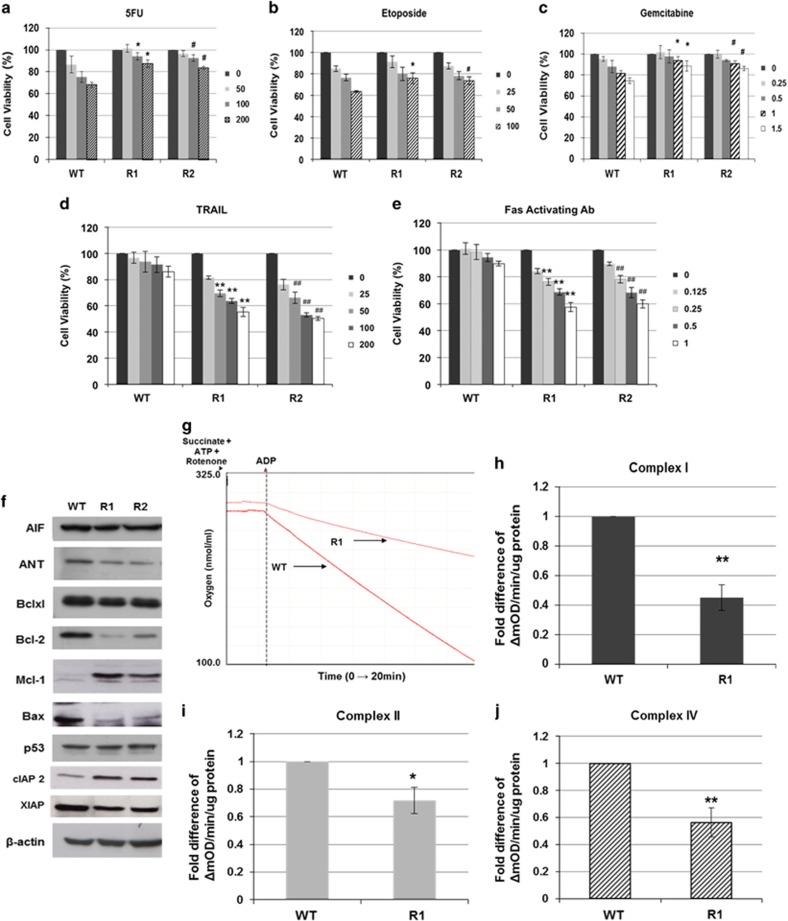
Cisplatin-resistant cells exhibit cross-resistance to DNA-damaging agents but not death-receptor signaling. A549 WT and the cisplatin-resistant cells were treated with (**a**) 5-fluorouracil (*μ*M), (**b**) etoposide (*μ*M), (**c**) gemcitabine (mM), (**d**) TRAIL (ng/ml) or (**e**) Fas activating antibody (*μ*g/ml) at the indicated doses for 24 h. Cell viability was determined by MTT assay and expressed as % of untreated control. *^,#^*P*<0.05 and **^,##^*P*<0.005 compared with WT treated at respective dose. (**f**) Western blot analysis of various proteins important in regulating mitochondria-mediated apoptotic signaling. β-Actin was used as a loading control. (**g**) Oxygen consumption of WT and R1 cells were assessed using a Clark electrode. State 3 respiration was initiated with addition of exogenous 0.2 mM ADP (arrow). The slope of the curve is a measure of the rate of oxygen consumption for a period of 20 min. Respiratory ETC complex I (**h)**, II (**i)** and IV (**j**) activities were measured in WT and R1 cells using the 96-well enzymatic-based microplate assay kit. *P<0.05 and **P<0.005 compared with WT control. ΔmOD indicates change in maximum OD at 340nm. Data shown are mean±S.D. of at least three independent experiments

To investigate whether R1 and R2 cells were also resistant to other nonplatinum-based compounds, we screened the cells using a series of conventional therapeutic agents. Indeed, R1 and R2 cells exhibited cross-resistance to other DNA-damaging agents such as 5-Fluorouracil (5-FU), gemcitabine and etoposide (Figures 2a–c). The efficacy of these agents is dependent upon efficient intrinsic death signaling. Interestingly, with the exception of anti-apoptotic Bcl-2 protein, other components of the intrinsic apoptotic pathway appeared to be rewired to limit death execution via the mitochondria in R1 and R2 cells; Bax and ANT were markedly downregulated, whereas the anti-apoptotic proteins, cIAP2 (cellular inhibitor of apoptosis protein 2) and Mcl-1 (myeloid cell leukemia sequence 1), were upregulated in R1 and R2 cells (Figure 2f). There was no significant difference in the expression of Apaf-1, caspase 8 and Bak in the three cell lines (Supplementary Figure S1E). Moreover, mitochondrial respiratory activity was significantly lower in cisplatin-resistant cells; mitochondria oxygen consumption and activity of complex I, II and IV were clearly reduced in R1 cells (Figures 2g–j). These results suggest a possible adaptive response that may render cisplatin-resistant cells refractory to mitochondria-driven execution. Interestingly, R1 cells exhibited enhanced sensitivity to 2-deoxyglucose (2DG) (Supplementary Figure S1B), indicating a possible switch from oxidative phosphorylation to a Warburg phenotype in cisplatin-resistant cells.

### Remarkable upregulation of death receptor sensitivity in cisplatin-resistant cells

**Figure 3:**
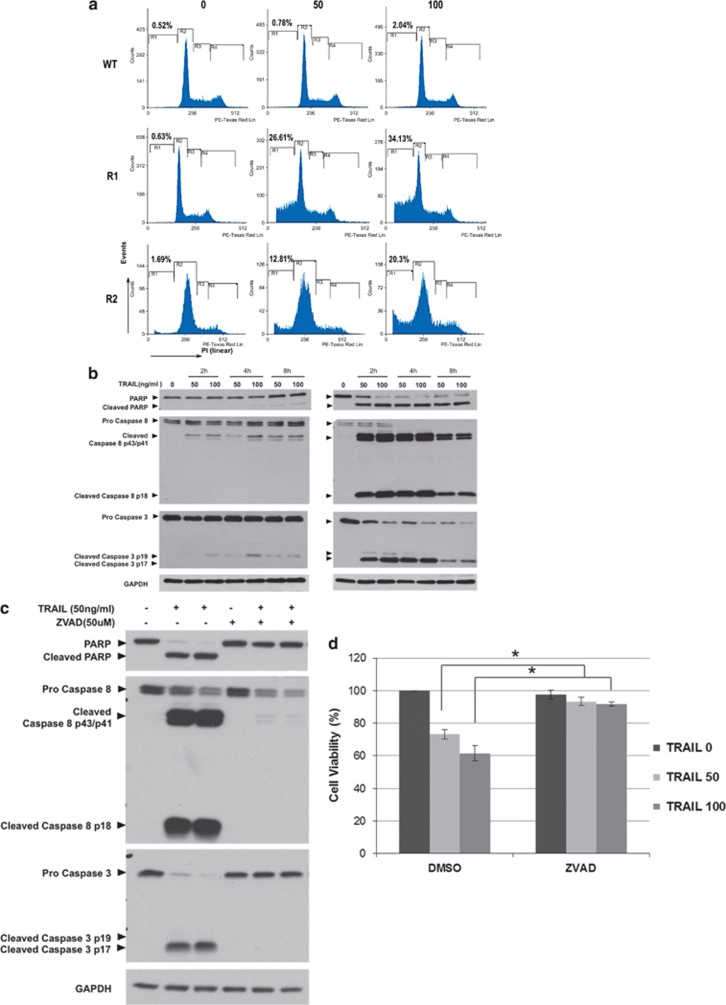
TRAIL induces caspase-dependent apoptosis in cisplatin-resistant cells. (**a**) Cell cycle analysis. Cells (A549 WT, R1 and R2) were treated with increasing doses of TRAIL for 4 h and DNA content was analyzed by flow cytometry after staining with propidium iodide (PI); y axis: events, x axis: PI linear fluorescence. (**b**) A549 WT and R1 cells were treated with the indicated doses of TRAIL in a time-dependent manner. Western blotting for caspase 3 and caspase 8 processing as well as PARP cleavage was performed using GAPDH as the loading control. (**c**) Cisplatin-resistant R1 cells were preincubated with ZVAD-fmk (50 *μ*M) for 1 h followed by 24 h of treatment with 50 ng/ml of TRAIL. Whole-cell lysates were subjected to western blotting for caspase 3 and caspase 8 processing as well as PARP cleavage. GAPDH was used as loading control. (**d**) R1 cells were treated with 50 or 100 ng/ml of TRAIL in the presence or absence of 50 *μ*M ZVAD-fmk and cell viability was measured by MTT assay and expressed as % of untreated cells. Data shown are mean±S.D. of at least three independent experiments. *P-value<0.05 compared to cells treated with TRAIL alone

Despite cross-resistance to triggers that rely on intrinsic apoptotic pathway, R1 and R2 cells exhibited remarkable sensitivity to death receptor ligation such as Fas-activating antibody or TRAIL (Figures 2d and e). Indeed, exposure of R1 and R2 cells to TRAIL resulted in a dose-dependent increase in DNA fragmentation, caspase 8 and 3 processing and PARP cleavage (Figures 3a and b and Supplementary Figure S2A), phosphatidylserine (PS) exposure and activation of caspases (Supplementary Figures S2B and C). Furthermore, the pan-caspase inhibitor ZVAD-fmk (carbobenzoxy-valyl-alanyl-aspartyl-[O-methyl]- fluoromethylketone) (Figure 3c) abrogated TRAIL-induced cell death in R1 cells (Figure 3d), thus corroborating the crucial involvement of caspase 8 and 3 in death receptor-induced execution of cisplatin-resistant cells. The dominant role of caspase 8 in the execution signal was also confirmed by the ability of the caspase 8-specific tetrapeptide inhibitor (Z-IETD-fmk) to completely rescue R1 cells from TRAIL-induced apoptosis (Supplementary Figure S2D). Furthermore, overexpression of Bcl-2 did not affect sensitivity of R1 or R2 cells to TRAIL (Supplementary Figure S1C), suggesting a mechanism independent of mitochondria.

**Figure 4:**
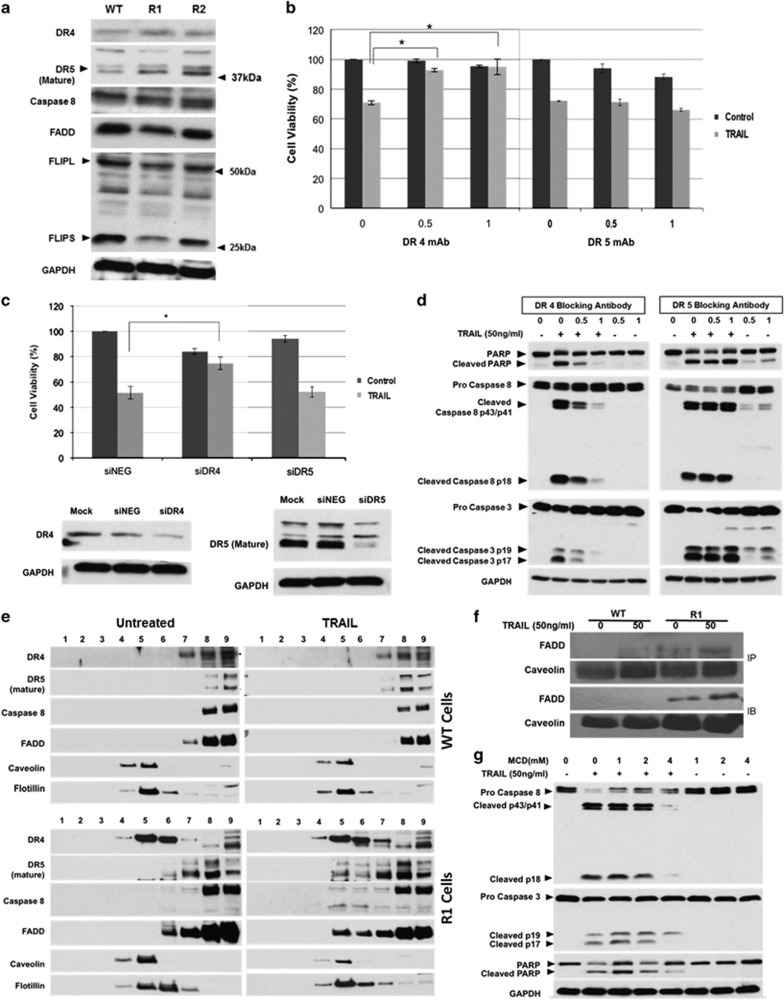
DR4 is redistributed to lipid raft subdomains in R1 cells. (**a**) Western blot analysis for various proteins important in regulating the extrinsic apoptotic pathway. GAPDH was used as loading control. (**b**) R1 cells were preincubated with soluble monoclonal antibodies against DR4 or DR5 (*μ*g) for 1 h followed by 24 h of treatment with 50 ng/ml of TRAIL. Cell viability was determined by MTT assay and expressed as % of untreated control. (**c**) R1 cells were transiently transfected with either scrambled siRNA or siRNA against DR4 or DR5 for 48 h followed by 50 ng/ml of TRAIL treatment for 24 h and cell viability were determined using MTT assay; *P<0.05. (**d**) R1 cells were preincubated with blocking antibodies against DR4 and DR5 for 1 h followed by 24 h of treatment with 50 ng/ml of TRAIL and lysates were subjected to western blot analysis for the assessment of caspase 3 and caspase 8 processing as well as PARP cleavage. (**e**) WT (top) and R1 cells (bottom) were treated with 50 ng/ml TRAIL for 15 min and subjected to discontinuous sucrose density gradients of Triton X-100 cell lysates for separation of lipid raft and non-raft fractions. One to 9 fractions were examined by western blots for the presence of DR4 and DR5 (first two rows). Lipid raft fractions 5 and 6 were identified by western blots. Flotillin and caveolin-1 were used as lipid raft markers. (**f**) Following TRAIL (50 ng/ml) treatment for 15 min, WT and R1 cells were subjected to discontinuous sucrose density gradients of Triton X-100 cell lysates for separation of lipid raft and nonraft fractions. Fractions 4–6 were collected and immunoprecipitated with anti-caveolin-1 antibody. As shown, caveolin-1 and FADD protein expression were detected by immunoblotting. (**g**) R1 cells were preincubated with the indicated doses of MCD for 1 h followed by 24 h of treatment with 50 ng/ml of TRAIL. Whole-cell lysates were subjected to western blot analysis for the assessment of caspase 3 and caspase 8 processing as well as PARP cleavage. GAPDH was used as loading control. Data shown are mean±S.D. of at least three independent experiments

Ligation of death receptors induces the assembly of a death-inducing signaling complex (DISC) that comprises pro-caspase 8 and the adaptor protein FADD (Fas-associated death domain-containing protein). Interestingly, DR5 (TRAIL-R2) expression was clearly augmented in R1 and R2 cells, whereas a moderate increase in DR4 (TRAIL-R1) level was observed (Figure 4a). However, no difference in the cell surface expression of DR4 or DR5 was detected in the three cell lines (Supplementary Figure S3B). In addition, the expression of pro-caspase 8 or FADD was similar across WT and cisplatin-resistant cells, and the DISC inhibitory protein cFLIP (cellular FLICE-like inhibitory protein) was downregulated in the cisplatin-resistant clones, particularly in R1 cells (Figure 4a). Notably, TRAIL induced a significant downregulation of cFLIP, XIAP and cIAP2 in R1 and R2 cells as compared with WT A549 cells (Supplementary Figure S3A).

Intriguingly, blocking of DR4, but not DR5, abrogated TRAIL-induced cell death in R1 cells (Figure 4b). Similarly, small interfering RNA (siRNA)-mediated downregulation of DR4 (but not DR5) virtually completely rescued R1 cells from TRAIL-induced cell death (Figure 4c). In addition, unlike anti-DR5, DR4 blocking antibody inhibited caspase 8 and 3 activation and PARP cleavage (Figure 4d). These results indicate that DR4, but not DR5, plays a critical role in TRAIL-mediated cell death in cisplatin-resistant cells.

### Redistribution of DR4 to the lipid rafts sensitizes cisplatin-resistant R1 cells to TRAIL-induced apoptosis

So far, the results provide evidence that the increased sensitivity of cisplatin-resistant R1 cells to TRAIL is a function of DR4 signaling. Of note, redistribution of death receptors to lipid rafts subdomains within the plasma membrane have been shown to amplify death receptor signaling.^17, 18^ Intrigued by this, we hypothesized that the increased sensitivity of cisplatin-resistant cells to TRAIL could be a function of differential localization of DR4 (*versus* DR5) to lipid raft subdomains. Using sucrose gradient density centrifugation to isolate lipid raft subdomains and two raft-associated proteins, caveolin and flotillin, as markers, results indicate that DR4 and FADD colocalized with the same fractions as caveolin and flotillin in R1 cells even in the absence of TRAIL (Figures 4e and f). A similar distribution for Fas (CD95) was observed that was further reinforced upon ligation of the Fas (CD95) receptor (Supplementary Figure S3C). Of note, neither DR4 nor DR5 localized to the raft fractions in WT cells with or without TRAIL (Figure 4e). Notably, exposure to TRAIL resulted in the recruitment of pro-caspase 8 and FADD to the lipid rafts in R1 cells (Figures 4e and f). These data were corroborated by immunofluorescence analysis demonstrating that DR4 (green) and caveolin (red) were colocalized in R1 cells even in the absence of TRAIL (Supplementary Figures S4A and B). Quantitative analysis using Pearson's correlation coefficient revealed a significant recruitment of DR4 in R1 cells as compared with WT cell (Supplementary Figure S4F). Notably, caspase 8 (green) was shown to colocalize with caveolin (red) after TRAIL exposure in R1 cells but not in the WT cells (Supplementary Figure S4C and D). Furthermore, the lipid raft disruptor, methylcyclodextrin-*β* (MCD), blocked TRAIL-induced caspase activation and PARP cleavage in R1 cells (Figure 4g) by disrupting the localization of DR4 in the lipid rafts (Supplementary Figure S4E). These data indicate that DR4 aggregation at the lipid rafts is responsible for the enhanced sensitivity of cisplatin-resistant cells to death receptor signaling.

### TRAIL-induced cell death in cisplatin-resistant R1 cells involves the generation of reactive nitrogen species

**Figure 5:**
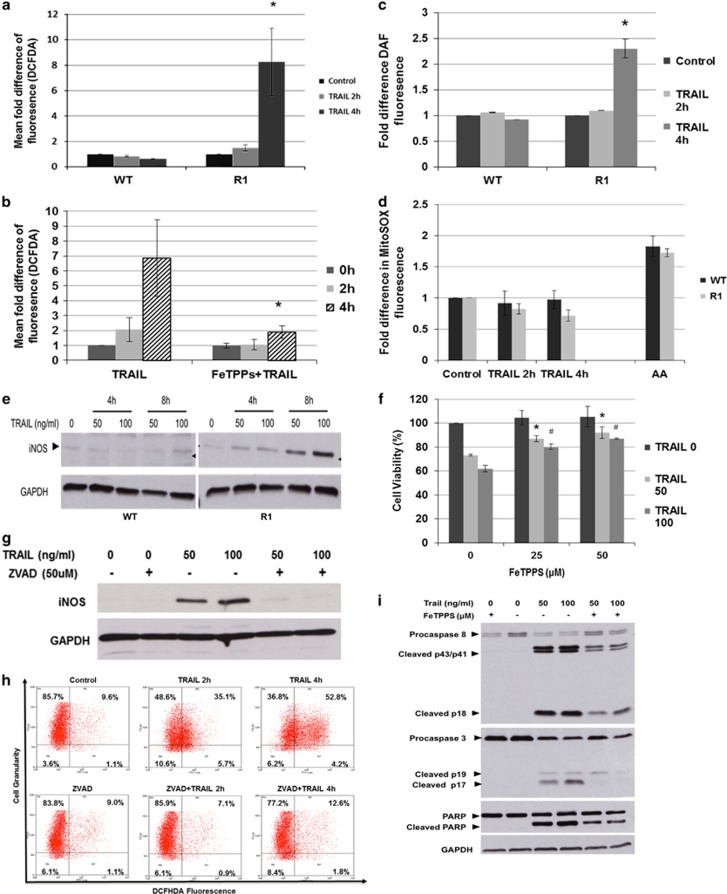
TRAIL-induced cell death in R1 cells involves the generation of reactive nitrogen species. (**a**) WT and R1 cells were treated with 50 ng/ml of TRAIL for 2 and 4 h. Cells were subsequently harvested and analyzed by flow cytometry for ROS production using redox-sensitive probe DCFH-DA. (**b**) R1 cells were preincubated with 50 *μ*M FeTPPs for 1 h followed by 50 ng/ml of TRAIL for 2 or 4 h. Cells were subsequently harvested and analyzed by flow cytometry after loading with DCFH-DA. (**c**) Cells were treated as above and loaded with NO-specific probe DAF or (**d**) MitoSox before flow cytomteric analysis. Antimycin A (AA) was used as a positive control for mitochondrial O_2_^−^. Data are shown as mean±S.D. of fold differences of fluorescence from untreated cells for at least three independent experiments. (**e**) Western blot analysis of iNOS following treatment of WT and R1 cells with 50 and 100 ng/ml of TRAIL. (**f**) R1 cells were preincubated with FeTPPs (50 *μ*M) for 1 h followed by 24 h of treatment with 50 ng/ml of TRAIL. Cell viability was determined by MTT assay and expressed as % of untreated control cells. *P<0.05 compared with 50 ng/ml of TRAIL alone and ^#^*P*<0.05 compared with 100 ng/ml of TRAIL alone. Data shown are mean±S.D. of at least three independent experiments. (**g**) Western blot analysis of iNOS in R1 cells, pretreated with 50 *μ*M ZVAD for 1 h followed by 50 ng/ml of TRAIL for 8 h. (**h**) WT and R1 cells were pretreated with 50 *μ*M ZVAD for 1 h followed by 50 ng/ml of TRAIL for 2 and 4 h. Cells were harvested and analyzed by flow cytometry for intracellular ROS production with DCFH-DA. Data shown are representative of at least three independent experiments. (**i**) R1 cells were preincubated with FeTPPs for 1 h followed by 24 h of treatment with 50 ng/ml of TRAIL and whole-cell lysates were subjected to western blot analysis for the assessment of caspase 3 and 8 processing as well as PARP cleavage. GAPDH was used as loading control

Reactive oxygen species (ROS) and reactive nitrogen species (RNS) are known mediators of death receptor signaling.^19, 20, 21^ In addition, our previous work has highlighted the role of intracellular ROS in drug-induced sensitization to TRAIL.^22^ Thus, we investigated the involvement of ROS/RNS in the heightened sensitivity of R1 cells to TRAIL. Using a fluorescence probe (DCFH-DA) that primarily detects hydrogen peroxide (H_2_O_2_) and peroxynitrite (ONOO^−^), we found a marked increase in DCF fluorescence in TRAIL-treated R1 cells, compared with WT cells (Figure 5a and Supplementary Figure S5A). To ascertain the ROS/RNS species involved in TRAIL signaling, we employed two antioxidants, FeTPPS (5,10,15,20-Tetrakis(4-sulfonatophenyl)porphyrinato iron (III), chloride) and catalase, that scavenge ONOO^−^ and H_2_O_2_ respectively. Interestingly, FeTPPS pretreatment blocked the increase in DCF fluorescence signal in TRAIL-treated R1 cells (Figure 5b), whereas catalase pretreatment neither blocked DCF fluorescence nor rescued cells from TRAIL-induced death (Supplementary Figures S5B and C). These data provide evidence to implicate ONOO^−^ in TRAIL-mediated execution of R1 cells.

ONOO^−^ is a reactive product generated from the reaction between nitric oxide (NO) and superoxide (O_2_^−^). To validate ONOO^−^ generation upon TRAIL treatment in R1 cells, we assessed intracellular NO and O_2_^−^ using the fluorescent probes, 4-amino-5-methylamino-2′,7′-difluorofluorescein diacetate (DAF-FM; for NO) and MitoSOX (for mitochondrial O_2_^−^). Whereas intracellular NO was elevated in TRAIL-treated R1 cells (Figure 5c and Supplementary Figure S6A), the converse was true for mitochondrial O_2_^−^ (Figure 5d). Based on these data, it is likely that NO generated upon TRAIL treatment reacts with endogenous mitochondrial O_2_^−^ to produce ONOO^−^, as evidenced by the decline in MitoSOX fluorescence in R1 cells. Indeed, the level of inducible nitric oxide synthase (iNOS; one of the most common inducible sources of intracellular NO) was upregulated in TRAIL-treated R1 cells (Figure 5e), lending further support to the involvement of NO and subsequently ONOO^−^.

In order to establish that ONOO^−^ was involved in TRAIL signaling in R1 cells, we assessed the viability of TRAIL-treated R1 cells after FeTPPS pretreatment. Indeed, scavenging ONOO^−^ by *a priori* treatment with FeTPPS significantly rescued R1 cells from TRAIL-induced cell death (Figure 5f) as well as blocked the effect on tumor long-term colony-forming ability (Supplementary Figure S6C). ONOO^−^ may be a downstream product of caspase activation or an upstream activator of caspases. To address this, we tested whether ZVAD-fmk and FeTPPS could inhibit TRAIL-induced ONOO^−^ production or caspase activation, respectively. In this regard, ZVAD-fmk completely blocked TRAIL-mediated induction of iNOS and ONOO^−^ production (Figures 5g and h). Surprisingly, preincubation with FeTPPS also partially inhibited TRAIL-induced caspase 8/3 activation (Figure 5i). These data suggest the presence of a potential positive feedback loop, with iNOS and ONOO^−^ production situated downstream of caspase 8/3 activation, whereas ONOO^−^, once produced, could further amplify the activation of caspases and thereby augment the death-inducing ability of TRAIL in cisplatin-resistant R1 cells.

### TRAIL sensitivity is not exclusive to cisplatin-resistant clones of A549 cell line

R1 and R2 are homogenous clones that were selected and developed from a single cisplatin-resistant NSCLC A549 cell mix. In our search for cell models that are representative of a heterogeneous tumor environment, we developed two other cisplatin-resistant heterogeneous cell lines from an ovarian carcinoma model, A2780, as well as another NSCLC model, H2030 cells.

Akin to R1/R2 cells, cisplatin-resistant A2780 (termed A2780CR) and H2030 cells (termed H2030CR) exhibited a sparse and disorganized morphology as compared with their WT counterparts (Supplementary Figures S7A and B). A2790CR and H2030CR cells also elicited resistance to cisplatin-induced cell death, caspase 8, 9 and 3 activation and PARP cleavage (Supplementary Figures S8A–D). Importantly, the two heterogeneous cisplatin-resistant cell lines were particularly sensitive to TRAIL treatment. Compared with their WT counterparts, TRAIL treatment resulted in a significant loss in viability (Supplementary Figures S8E and F), activation of caspases 8, 9 and 3 and PARP cleavage in A2790CR and H2030CR cells (Supplementary Figures S8G and H). These data demonstrate that TRAIL sensitivity is likely a general phenomenon in cisplatin-resistant cells and not restricted to a particular cell type.

### Synergistic effect of TRAIL in combination with cisplatin in the treatment of cisplatin-resistant cells

**Figure 6:**
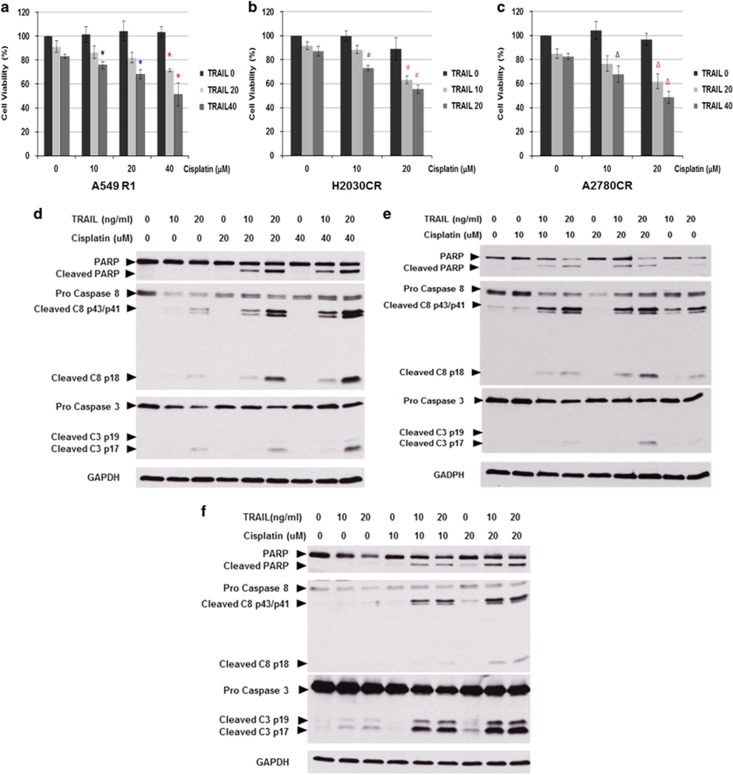
TRAIL exposure restores apoptosis sensitivity of cisplatin-resistant (CR) cells. (**a**) A549 R1, (**b**) H2030 CR and (**c**) A2780 CR cells were pretreated with varying doses of TRAIL for 2 h and followed with the indicated doses of cisplatin for 24 h. Cell viability was assessed by MTT and expressed as % of untreated control cells. Data shown are mean±S.D. of at least three independent experiments. Concurrently, the cell lysates from (**d**) A549 R1, (**e**) H2030 CR and (**f**) A2780 CR were collected to assay for caspase 3 and caspase 8 processing as well as PARP cleavage by western blot analysis. GAPDH was used as loading control. *, #, Δ indicated *P*-Value<0.05 compared to cells treated with cisplatin alone

Having observed a remarkable upregulation of death receptor signaling in cancer cells rendered resistant to cisplatin, we explored the potential use of death receptor ligation in combination with cisplatin as a novel therapeutic strategy to combat recalcitrant tumors. Interestingly, a combination of low dose of cisplatin and TRAIL was significantly more potent against the three cisplatin-resistant cell lines as compared with the single-agent treatment (Figures 6a–c). In agreement with these findings, combinatory treatment of cisplatin and TRAIL markedly enhanced the activities of caspases 8, 9 and 3 in the three cisplatin-resistant cell lines (Figures 6d–f). Taken together, these data highlight the potential of exploiting the enhanced sensitivity to death receptor-induced apoptosis as a therapeutic strategy against tumors that are refractory to platinum-based compounds.

## Discussion

Cisplatin triggers DNA damage response and p53-mediated transcription of pro-apoptotic proteins such as p21, PUMA, and Bax, leading to mitochondria-driven cell death.^23, 24, 25^ The effectiveness of cisplatin is hampered by undesirable adverse effects as well as the development of drug resistance over time. Among the most commonly described mechanism of cisplatin resistance is the ability of cancer cells to evade the mitochondrial apoptotic response induced by cisplatin. Alterations in mitochondrial physiology and function have been reported by others in cisplatin-resistant cells.^26, 27, 28^ Similarly, we detected significant changes in the mitochondria of cisplatin-resistant cell lines. Mitochondria respiratory activity was significantly suppressed in R1 cells as compared with WT cells (Figures 2g–j). Of note, pro-apoptotic proteins Bax and ANT were downregulated, whereas anti-apoptotic Mcl-1 and cIAP2 were elevated in cisplatin-resistant cells (Figure 2f). Whereas Bcl-2 was paradoxically downregulated in R1 and R2 cells, this was compensated by the marked increase in Mcl-1, corroborating an earlier report indicating Mcl-1 dependency of cisplatin-resistant cancer cells.^29^ Moreover, R1 cells undergo a switch from oxidative phosphorylation to a Warburg phenotype. It is noteworthy that not only changes to the mitochondria, but also a significant remodeling of the ER proteome, in particular a remarkable upregulation of protein disulfide isomerases PDIA4 and PDIA6, was recently reported in cisplatin-resistant A549 cells.^30^

When continuously exposed to a single class of chemotherapeutic compound, tumors may gain resistance through the rewiring of oncogenic signaling networks. Such perturbation in cellular signaling, however, could potentially alter and potentiate other execution pathways as well. In this regard, we demonstrate that cisplatin-resistant cancer cell lines that have acquired the ability to evade the mitochondria apoptotic machinery are particularly sensitive to death receptor-mediated apoptosis. As compared with WT cells, cisplatin-resistant cell lines generated from both NSCLC and ovarian cancer cells were exceptionally sensitive to TRAIL- and Fas-activating antibody-induced apoptosis. The augmented sensitivity to TRAIL resulted from the redistribution of DR4 (but not DR5) and the various DISC components to lipid raft subdomains of the plasma membrane in cisplatin-resistant cells. Preligation congregation of DR4 in lipid rafts could have facilitated the assembly and activation of DISC upon TRAIL ligation. Indeed, this observation is also supported by previous evidence demonstrating that the distribution of death receptors and DISC components to the lipid rafts could greatly promote death receptor-mediated apoptosis, whereas non-raft receptors in turn inhibit caspase 8 activation.^17, 18, 31^

In addition to the canonical death receptor signaling (DNA fragmentation and caspase activation), TRAIL also augmented the expression of iNOS, exclusively in cisplatin-resistant cells. This resulted in increased NO production that then reacted with O_2_^−^ to produce the highly reactive ONOO^−^. Catalytic degradation of ONOO^−^ blocked the death-inducing effect of TRAIL, indicating that ONOO^−^ is required for maximal death execution by TRAIL in cisplatin-resistant cells. Our observations are supported by previous evidence demonstrating that the introduction of NO donors or the overexpression of NOS could sensitize cells to Fas signaling,^32, 33, 34^ whereas direct treatment of ONOO^−^ itself could also enhance Fas-induced apoptosis in leukemia cells.^35^ Interestingly, we found that ONOO^−^ production and caspase activation induced by TRAIL in cisplatin-resistant R1 cells were interdependent events. On one hand, inhibition of caspase activation by ZVAD-fmk abrogated TRAIL-induced iNOS expression and ONOO^−^ production, and on the other hand, scavenging ONOO^−^ with FeTPPS also blocked, although incompletely, the proteolytic cleavage of caspase 8 and 3. These data indicate the presence of a positive feedback loop upon TRAIL treatment in cisplatin-resistant cells, whereby the activation of caspases by death receptors induced an initial increase in iNOS expression and ONOO^−^ production that could in turn amplify death execution by further promoting proteolytic processing of caspases. These results point to ONOO^−^ as a critical factor in positive feedback amplification of death receptor signaling in cancer cells rendered resistant to drug-induced apoptosis. The mechanism(s) on how ONOO^−^ could stimulate the proteolytic cleavage of caspases is unclear and warrant(s) further investigations.

The combinatory usage of TRAIL with conventional chemotherapeutic agents has been rather promising in preclinical settings. When combined with TRAIL, synergistic effects have been reported for a wide variety of cytotoxic compounds including 5-FU, gemcitabine, doxorubicin and paclitaxel.^36, 37, 38, 39^ Here, our work further accentuates the potential of death receptor ligation as a second-line therapeutic agent against recalcitrant malignancies. We provide novel evidence that cancer cells that have acquired resistance to platinum-based compounds are resensitized to cisplatin by *a priori* exposure to TRAIL (Figure 6a). Notably, the sensitization to death receptor-induced apoptosis appears to be a general phenomenon affecting more than a single clone of cisplatin-resistant cells; heterogeneous cisplatin-resistant clones derived from NSCLC and ovarian cancer are equally susceptible to such resensitization properties of TRAIL (Supplementary Figure S8). The efficacy of death receptor ligation in combination with cisplatin in the treatment of cisplatin-resistant malignancies is evident, and should therefore be considered as a second-line therapeutic strategy for recalcitrant malignancies. A similar synergistic effect of TRAIL and cisplatin was reported in human head and neck squamous cell carcinoma cells.^40^

TRAIL has a superior clinical safety profile as compared with other conventional chemotherapeutic agents. In a phase Ia study, TRAIL was reported to be safe and well tolerated up to a dose of 15 mg/kg with no drug-related dose-limiting toxicities observed and only minor cases of adverse events (fatigue and anorexia).^41^ Notwithstanding its promising safety profile, the clinical efficacy of TRAIL in the treatment of NSCLC remains doubtful. A promising treatment response rate was first reported in recurrent or advanced NSCLC patients treated with TRAIL in combination with paclitaxel, carboplatin and bevacizumab (PCB) in a phase Ib study. However, the potential of TRAIL as a first-line therapy for advanced NSCLC was greatly dampened when a subsequent randomized phase II study reported little, if at all, improvement in overall patient response rate when TRAIL was included into conventional PCB treatment regimen.^42^ Despite these setbacks, the potential of death receptor ligation as a chemotherapeutic agent should not be completely disregarded. Evidence from our work indicates that TRAIL is particularly efficacious against cisplatin-resistant clones but not the parental cell lines. Discrepancies in overall response rate between the phase Ib and phase II trials may have resulted from the variation in patient disease conditions between the trials. It is plausible that patients with recurrent NSCLC may have responded better compared with patients who were diagnosed with advanced NSCLC without prior PCB treatment. As such, we propose that TRAIL or antibody-mediated ligation of death receptors, in combination with PCB, could have therapeutic potential as a second-line treatment for recurrent/relapsed tumors rather than as a first-line regiment for advanced malignancies.

## Materials and Methods

### Cell culture and generation of cisplatin-resistant variants

The human NSCLC A549 (WT, R1 and R2) cells were cultured in DMEM/F12 supplemented with 10% fetal bovine serum, 1% L-glutamine and 1% penicillin/streptomycin. The ovarian A2780 WT and A2780 cisplatin-resistant cells were cultured in RPMI-1640 supplemented with 10% fetal bovine serum, 1% L-glutamine and 1% penicillin/streptomycin. The NSCLC H2030 cells were purchased from American Type Cell Culture (ATCC, Rockville, MD, USA) and cultured in RPMI-1640 supplemented with 10% fetal bovine serum, 1% L-glutamine and 1% penicillin/streptomycin. All cell lines used were grown and passaged in a humidified incubator at 37 °C with 5% CO_2_.

To generate cisplatin-resistant cells from NSCLC A549 cells, the parental A549 cells were subjected to a low sublethal dose of 5 *μ*M cisplatin (Sigma Aldrich, St Louis, MO, USA) consistently for a period of 6 months. Next, individual clones were isolated from a mix of heterogeneous cisplatin-resistant cells using cloning cylinders and thus establishing R1 and R2 cells. Cisplatin-resistant R1 and R2 cells were then subsequently cultured in the conditions as mentioned above. Similarly, for the generation of cisplatin-resistant cells from NSCLC H2030 and ovarian A2780, parental cells were treated with 5 *μ*M of cisplatin for a period of 6 months. The cisplatin-resistant cells were then subsequently maintained as heterogeneous cultures in conditions as mentioned above.

### MTT proliferation assay

Cell viability following the various drug treatments was determined by the 3-(4,5-dimethyl-2-thiazolyl)-2,5-diphenyltetrazolium bromide (MTT, Sigma Aldrich) assay, as described previously.^43^ Cell viability experiments were similarly performed with preincubation for 1 h with pan-caspase inhibitor ZVAD-fmk (Biomol, Enzo Life Sciences Inc., Farmingdale, NY, USA), FeTPPS (Cayman Chemical, Ann Arbor, MI, USA), death receptor blocking antibodies, (anti-DR4 or anti-DR5; Alexis Enzo Life Sciences, Inc., Farmingdale, NY, USA), methyl-*β*-cyclodextrin (Sigma Aldrich), before the addition of soluble human recombinant TRAIL (*BML-SE721*; Enzo Life Sciences, Inc.). It should be pointed out that FeTPPS does not interfere with the MTT assay (Supplementary Figure S6B).

### PI staining for DNA fragmentation analysis

Cells were treated accordingly, harvested and pelleted down by centrifugation. After washing twice with ice-cold 1 × PBS, cells were fixed with ice-cold 70% (v/v) ethanol, incubated for at least 1 h at −20 °C and then pelleted by centrifugation at 2 500 r.p.m. for 5 min at 4 °C. Pelleted cells were then washed twice with ice-cold 1 × PBS before being stained with 500 *μ*l of PI/RNase (10 *μ*g/ml PI, 250 *μ*g/ml RNaseA in 1 × PBS) solution for 30 min at 37 °C. A total of 10 000 events were analyzed for DNA content with flow cytometry (CyAn ADP, Beckman Coulter, Brea, CA, USA) with the excitation set at 550 nm and emission at 610 nm. Data collected were analyzed with the Summit 4.3 software (Fullerton, CA, USA).

### Isolation of membrane lipid rafts by sucrose density gradient ultracentrifugation

The membrane lipid raft subdomains were isolated by sucrose density gradient ultracentrifugation as described previously^44^ and detailed in the Supplementary Materials and Methods. Here, 0.5 ml fractions were collected from top to bottom (named 1–9). The fractions were subjected to sodium dodecyl sulfate–polyacrylamide gel electrophoresis (SDS-PAGE) and western blot analysis and lipid rafts were validated by the presence of lipid raft markers, caveolin and flotillin.

### RNAi-mediated transient knockdown of DR4 and DR5

A549 R1 cells (at 0.08 × 10^6^ cells/well) were plated in six-well plates for 24 h before transfection to obtain ∼40% confluence the next day, and medium was replaced with antibiotic-free DMEM/F12 for ∼2 h before commencing transfection. Transient transfections were performed using Dharmafect transfection reagent and 50 nM of DR4 siRNA or 50 nM of DR5 siRNA smartpool siRNA (Dharmacon GE Healthcare, Lafayette, CO, USA), dissolved in opti-MEM (Life Technologies Thermo Fisher Scientific, Carlsbad, CA, USA). The control cells were transfected with scrambled nontargeting siRNA. At 24 h after transfection, the media were replaced with complete DMEM/F12 and cells were incubated further for 48 h before any subsequent experiments.

### Determination of intracellular ROS levels by flow cytometry

ROS/RNS levels were determined by flow cytomtery using specific probes. CM-H2-DCFH-DA (5-(and-6)-chloromethyl-2,7-dichlorofluorescin diacetate) was used for the detection of general ROS/RNS (including H_2_O_2_ and ONOO^−^) with Ex/Em wavelengths set at 495/520 nm. MitoSOX Red (Molecular Probes, Eugene, OR, USA) was used for the detection of mitochondrial O_2_^−^ with Ex/Em wavelength set at 510/580 nm. DAF-FM Diacetate was used for the detection of NO at Ex/Em wavelength of 495/515 nm. All probes are obtained from Life Technologies Thermo Fisher Scientific. Experimental protocols were performed as described previously.^45, 46^ Briefly, cells were trypsinized, centrifuged at 2 000 r.p.m. and washed in 1 × PBS. Cells were then resuspended in 200 *μ*l FBS-free media and loaded with the specific redox probe and incubated at 37 °C for 15 min. Stained cells were washed again with 1 × PBS and analzsed by flow cytometry (CyAn ADP, Beckman Coulter) using their respective excitation and emission wavelengths. At least 10 000 events were analyzed. Data collected were analyzed with Summit 4.3 software.

### Measurement of mitochondrial oxygen consumption

Mitochondrial respiration of the A549 WT and R1 cells was measured using the Oxytherm Electrode (Hansatech Instruments, Norfolk, UK). WT or R1 cells (30 × 10^6^) at exponential growth were harvested and permeabilized with digitonin. Samples were normalized by protein quantification at the start of the experiment. Oxygen consumption measurement was performed as described elsewhere.^47, 48, 49^

### Enzymatic activities of mitochondrial respiratory complexes I, II and IV

Quantitative analyses of the mitochondrial complexes I, II and IV activities were performed using the Human Complex I, II and IV activity Microplate Assay Kit provided by MitoSciences (Eugene, OR, USA). Experiments were carried out according to the protocol provided by the manufacturer and the enzymatic reactions were assayed by measuring the change in absorbance using a TECAN spectrophotometer (Tecan Trading AG, Mannedorf, Switzerland).

### Western blot analysis

Cells were harvested by centrifugation at 1200 r.p.m. for 5 min at 4 °C, washed with PBS and suspended in RIPA buffer (50 mM Tris-HCl (pH 7.6), 150 mM NaCl, 1% NP40, 0.1% SDS and 0.5% sodium deoxycholate) containing protease inhibitor cocktail (Roche Lifesciences, Indianapolis, IN, USA). Samples were centrifuged at 14 000 r.p.m. for 15 min at 4 °C. The cell lysates were then separated by 10–15% SDS-PAGE and transferred to polymer of vinylidene fluoride (PVDF) membranes (Bio-Rad Laboratories, Hercules, CA, USA) and blocked with 5% non-fat milk in PBST (PBS with 0.5% Tween-20). Primary antibodies used include anti-p53, anti-caspase-3, anti-caspase-8, anti-Bax, anti-caspase-9, anti-XIAP, Rabbit polyclonal anti-PARP, anti-DR5 (all from Cell Signalling Technology, Inc., Danvers, MA, USA), anti-Mcl-1, anti-Bcl-2, anti-*β*-actin, anti-GAPDH, anti-ANT, anti-AIF, anti-Bcl-xL (all from Santa Cruz Biotechnologies, Inc., Santa Cruz, CA, USA), anti-cIAP2, anti-FADD, anti-iNOS (all from BD Biosciences, San Jose, CA, USA), anti-DR4 (Upstate, EMD Millipore Corporation, Billerica, MA, USA), anti-caveolin, anti-flotillin (Abcam, Cambridge, UK), anti-cFLIP (Alexis, Enzo Life Sciences). After incubation with HRP-conjugated secondary antibody (Pierce, Thermo Fisher Scientific, Inc., Rockford, IL, USA), protein expression was detected by a chemiluminescence reaction (EMD Millipore).

## Concluding remarks

**Figure 7:**
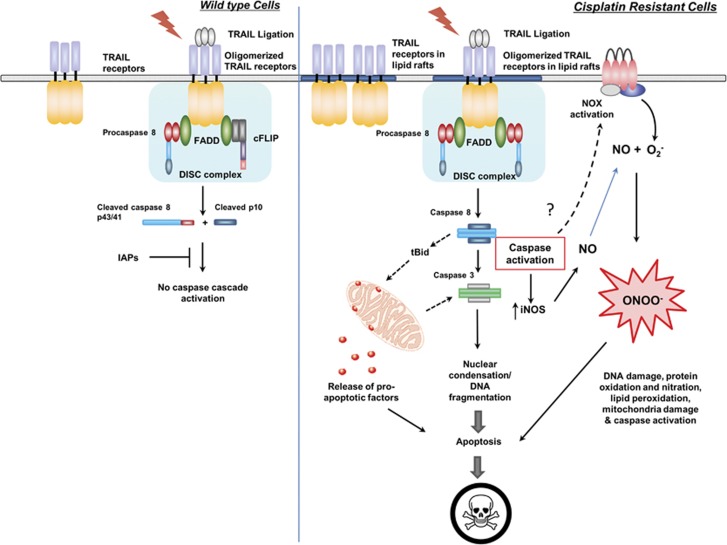
Schematic diagram of TRAIL sensitivity in WT and cisplatin-resistant cancer cells: in the WT cells, TRAIL receptors exist as monomers and upon TRAIL ligation, the receptors oligomerize to form the DISC and subsequently activation of pro-caspase 8. However, because of overexpression of anti-apoptotic proteins such as cFLIP, XIAP and cIAP2, apoptotic execution is imparied. In R1 cells, TRAIL receptors are redistributed to the lipid raft subdomian, and hence are ‘primed' for rapid activation. Upon ligation, the apoptotic signals are transduced promptly that involves downregulation of anti-apoptotic proteins as well as induction of an amplification signal via OONO^−^ formation for efficient death execution

We provide evidence for an amplification of death receptor signaling in cancer cells rendered resistant to cisplatin (Figure 7). This involves clustering of death receptors in lipid raft subdomains and their efficient activation upon exposure to the ligand resulting in downstream caspase activation. Our results also implicate intracellular ONOO^−^ as a critical trigger downstream of death receptor ligation. Of note, *a priori* exposure to TRAIL restored sensitivity of resistant cells to cisplatin. These data argue in favor of exploiting the enhanced sensitivity to death receptor-mediated apoptosis in the therapeutic management of tumors that have developed resistance to platinum-based drugs.

## Abbreviations

cFLIP: cellular FLICE-like inhibitory protein
cIAP2: cellular inhibitor of apoptosis protein 2
CM-H_2_DCFDA: 5-(and-6)-chloromethyl-2′,7′-dichlorofluorescin diacetate-dichlorofluorescein diacetate
DAF-FM: 4-amino-5-methylamino-2′,7′-difluorofluorescein
DISC: death-inducing signaling complex
DR4/5: death receptor 4/5
FADD: Fas-associated death domain-containing protein
FeTPPS: 5,10,15,20-Tetrakis(4-sulfonatophenyl)porphyrinato iron (III), chloride
H_2_O_2_: hydrogen peroxide
iNOS: inducible nitric oxide synthase
MCD-*β*: methyl-*β*-cyclodextrin
Mcl-1: myeloid cell leukemia sequence 1
NO: nitric oxide
NSCLC: non-small-cell lung cancer
O_2_^−^: superoxide anion
ONOO^−^: peroxynitrite
PARP: poly(ADP-ribose) polymerase
PI: propidium iodide
PS: phosphatidylserine
PVDF: polymer of vinylidene fluoride
RNS: reactive nitrogen species
ROS: reactive oxygen species
SDS: sodium dodecyl sulfate
SDS-PAGE: SDS-polyacrylamide gel electrophoresis
siRNA: small interfering RNA
SOD: superoxide dismutase
TNF: tumor necrosis factor
TRAIL: TNF-related apoptosis-inducing ligand
ZVAD-fmk: carbobenzoxy-valyl-alanyl-aspartyl-[O-methyl]- fluoromethylketone
