# Supplementary material for: Activation of surrogate death receptor signaling triggers peroxynitrite-dependent execution of cisplatin-resistant cancer cells
Source: Cell Death Dis. 2015 Oct 22;6(10):e1926–. doi: 10.1038/cddis.2015.299 (PMC4632318; doi:10.1038/cddis.2015.299)
Supplement: Supplementary Information [file cddis2015299x1.docx]

**Supplementary Materials and Methods**

***Colony forming assay***

Cells were treated with the drug for 24 h and at the end of drug treatment, cells were washed with 1 x PBS, trypisinized, and counted. For every treatment, 5,000 cells were re-plated onto a 100mm dish in complete tissue culture medium. Re-plated cells were left to form colonies over a period of 10 to 14 days in a humidified incubator at 37^o^C with 5% CO_2_, supplemented with complete medium every 3-5 days. Colonies formed were assessed by staining cells with crystal violet.

***Isolation of membrane lipid rafts by sucrose density gradient ultracentrifugation***

A549 WT cells (2x10^6^) or R1 cells (4x10^6^) were seeded in 100mm petri dishes 2 days before the lipid raft extractions. Cells were harvested and washed with ice-cold 1xPBS before incubating in 500ml of HEPES buffer (25 mM HEPES, 150 mM NaCl, pH7.4 containing 1% Triton X-100) and supplemented with the protease inhibitors (1mM PMSF, 10 μg/ml Aprotinin, 20 μg/ml Pepstatin A) and phosphatase inhibitors (1mM NaF, 1 mM Na_3_VO_4_) on ice for 10 min. Lysates were then subjected to 1 round of freeze-thaw action before homogenizing with a Dounce homogenizer for 20 strokes. The homogenized lysates were centrifuged at 500g for 10 min at 4°C to remove debris and the supernatants were subjected to 10 passes through a 27G syringe. This was followed by sonication (3 sets of 10 sec pulses at 40V) and protein quantification to standardize the amount of protein used for subsequent ultracentrifugation. The samples were then mixed with an equal volume of 80% sucrose (w/v) in HEPES buffer, transferred to a centrifuge tube and carefully overlaid with 2ml of 30% sucrose and 2ml of 5% sucrose solutions. The sucrose gradient was centrifuged for 21 h at 4ºC in a Beckman rotor at 32,000 rpm and nine 0.5 ml fractions were collected from top to bottom (named 1-9). The fractions were subjected to SDS-PAGE and Western blotting and lipid rafts are validated by the presence of lipid raft markers, caveolin-1 and flotillin.

***Detection of phosphatidylserine externalization by Annexin V staining***

Following treatment, cells were harvested and pelleted down by centrifugation at 2,000 rpm. Cells were washed in 1X PBS, then once in 1X Binding Buffer. Cells were then re-suspended in 1X Binding Buffer (diluted from 10X buffer: 0.1M Hepes, 1.4M NaCl, and 25 mM CaCl_2_ with dH_2_O at pH 7.4) and stained with FITC-conjugated Annexin V (*BD Biosciences; San Jose, CA, USA)* for 15 min at room temperature. Subsequently, cells were washed twice in 1X Binding Buffer and re-suspended in 200ul of 1x binding buffer for flow cytometry (Beckman Coulter Inc., Sunnyvale, CA) using an excitation wavelength of 488 and emission at 525nm. Data for 10,000 events were collected and analyzed by Summit 4.3 software.

***Determination of caspase activity***

Caspase activation was determined using synthetic fluorogenic oligopeptide substrates with the C-terminal aspartic acid modified with 7-amino 4 trifluoromethyl coumarin (AFC). Cells were harvested on ice at the end of different time points and lysed with chilled 1x Cell Lysis Buffer (*BD Pharmingen; San Diego, CA*). Protein concentration was quantified using the Coomassie Blue reagent. 5-10ug of cell lysates were added to equal volumes of 2X Reaction Buffer (20mM HEPES, 4mM EDTA, 10mM DTT and 3mM MgCl2 pH 7.4) and 1uM of respective caspase substrates was added on a 96-well plate; AcDEVD-AFC for caspase 3, Ac-VEID-AFC for caspase 6, Ac-IETD-AFC for caspase 8 and Ac-LEHD-AFC for caspase 9. Samples were incubated at 37°C for 1 hour, after which caspase activity was determined by measuring the relative AFC fluorescence with TECAN spectrophotometer (*Tecan Trading AG, Switzerland*) with excitation at 400nm and emission at 505nm. Protein concentration of each sample was determined using the Coomassie Blue reagent and caspase activity was normalized with protein amount and expressed as relative fluorescence unit (RFU)/μg protein.

# Analysis of DR4 and DR5 surface expression

A549 WT, R1 and R2 cells were seeded in 6-well plates for 24 h and harvested and washed with FACS buffer (1X PBS + 0.5% FBS) after detachment of adherent cells with EDTA. Cells were then stained with the phycoerythrin (PE)-conjugated mouse monoclonal anti-human DR4 or DR5 for 45 min at 4^o^C according to manufacturer’s instructions before washing and resuspension in FACS buffer for flow cytometry (*CyAn ADP, Beckman Coulter, USA*) using an excitation wavelength of 488. PE-conjugated mouse IgG2b was used as an isotype control and the obtained events were analysed by Summit 4.3 software.

***Confocal microscopy for lipid raft localization of DR4 and caspase-8***

A549 WT cells and R1 cells were plated in a 12-well plate on polylysine-coated coverslips. Cells were washed twice with 1X PBS and fixed with 2% paraformaldehyde in 1X PBS for 15min at room temperature. Next, cells were washed three times with ice cold 1X PBS and subjected to permeabilization with 50μM of digitonin in 1X PBS for 15min at room temperature. Cells were then washed three times with ice cold 1X PBS followed by 1h blocking with 5% BSA. Subsequently, cells were incubated with either mouse anti-DR4 or mouse anti-caspase-8 and rabbit anti-caveolin-1 in 1X PBS with 5% BSA (1:250 dilution for anti-DR-4 and anti-caspase-8, 1:500 dilution for anti-caveolin-1) for 2h. After that, they were washed three times with 1X PBS in 5% BSA followed by 1h incubation with 1:500 dilution for Alexafluor-488 anti-mouse secondary antibody (Invitrogen) and 1:500 dilution for Alexafluor-568 anti-rabbit secondary antibody in 1X PBS with 5% BSA. Cells were similarly washed three times with 1X PBS before the coverslips were mounted on glass slides and imaged with Olympus Fluoview FV1000 confocal microscope and images were analyzed using Olympus Fluoview 1.7 viewer.
