# Supplementary figures and images for: Activation of surrogate death receptor signaling triggers peroxynitrite-dependent execution of cisplatin-resistant cancer cells
Source: Cell Death Dis. 2015 Oct 22;6(10):e1926–. doi: 10.1038/cddis.2015.299 (PMC4632318; doi:10.1038/cddis.2015.299)

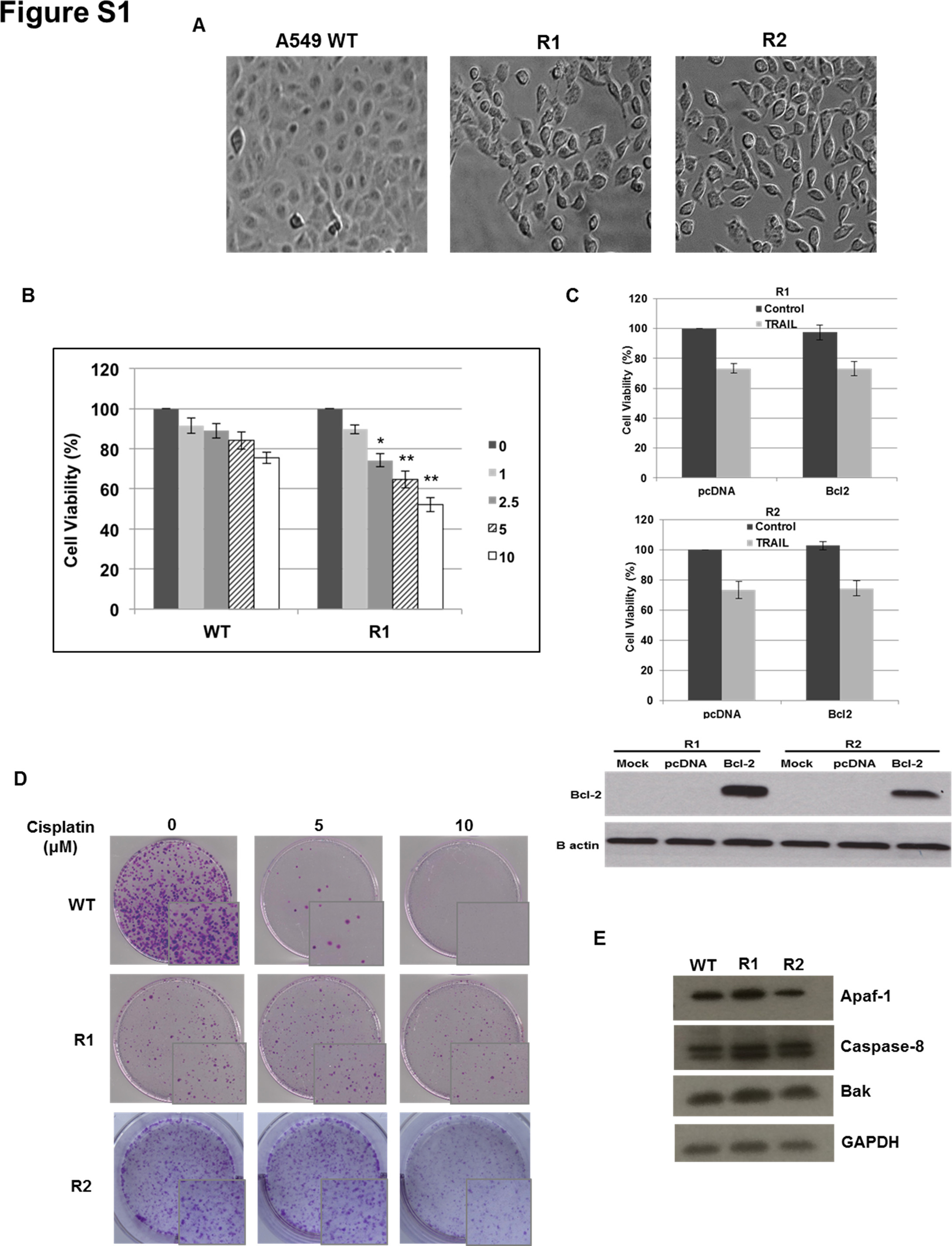

Supplement: Supplementary Figure S1 [file cddis2015299x2.tif]

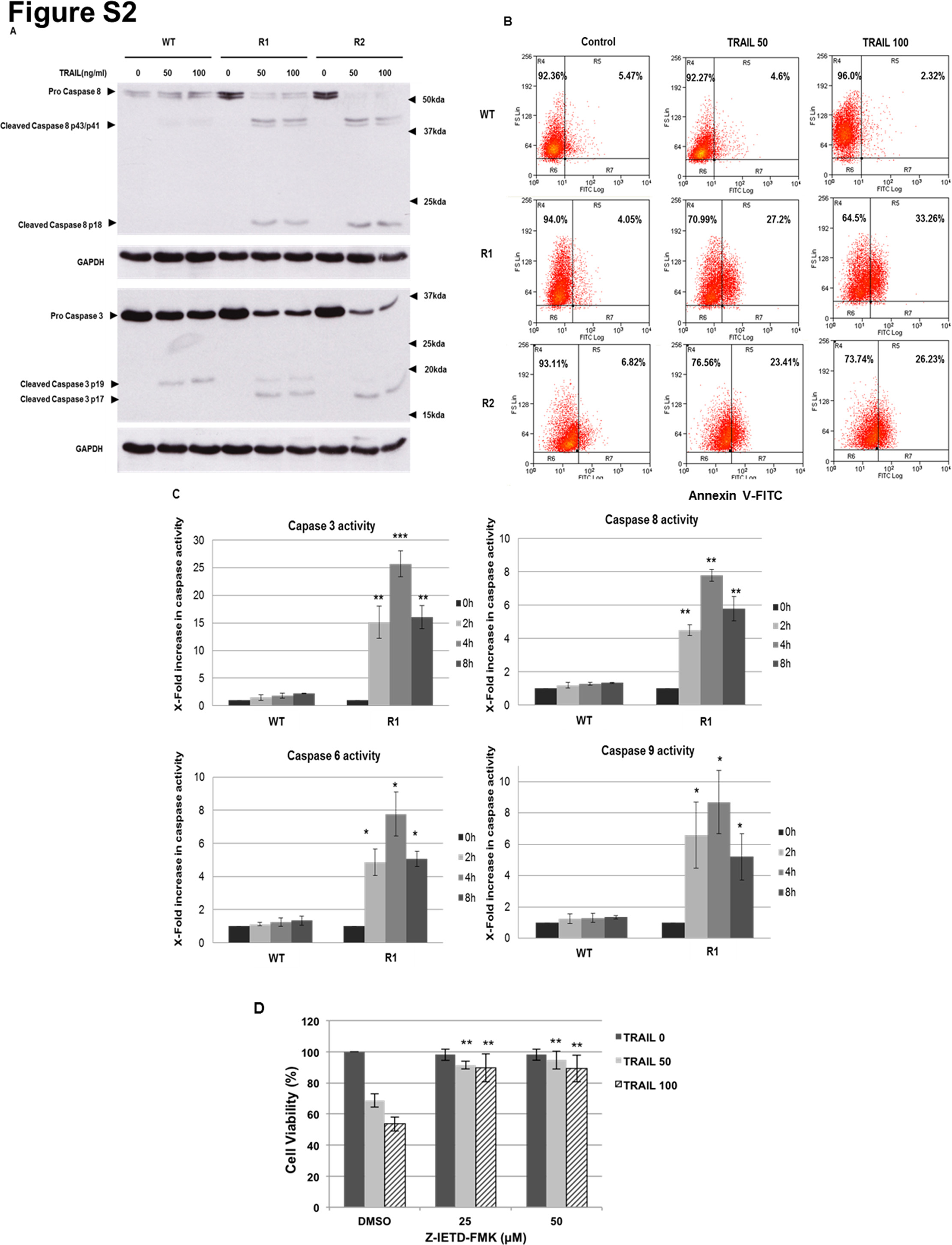

Supplement: Supplementary Figure S2 [file cddis2015299x3.tif]

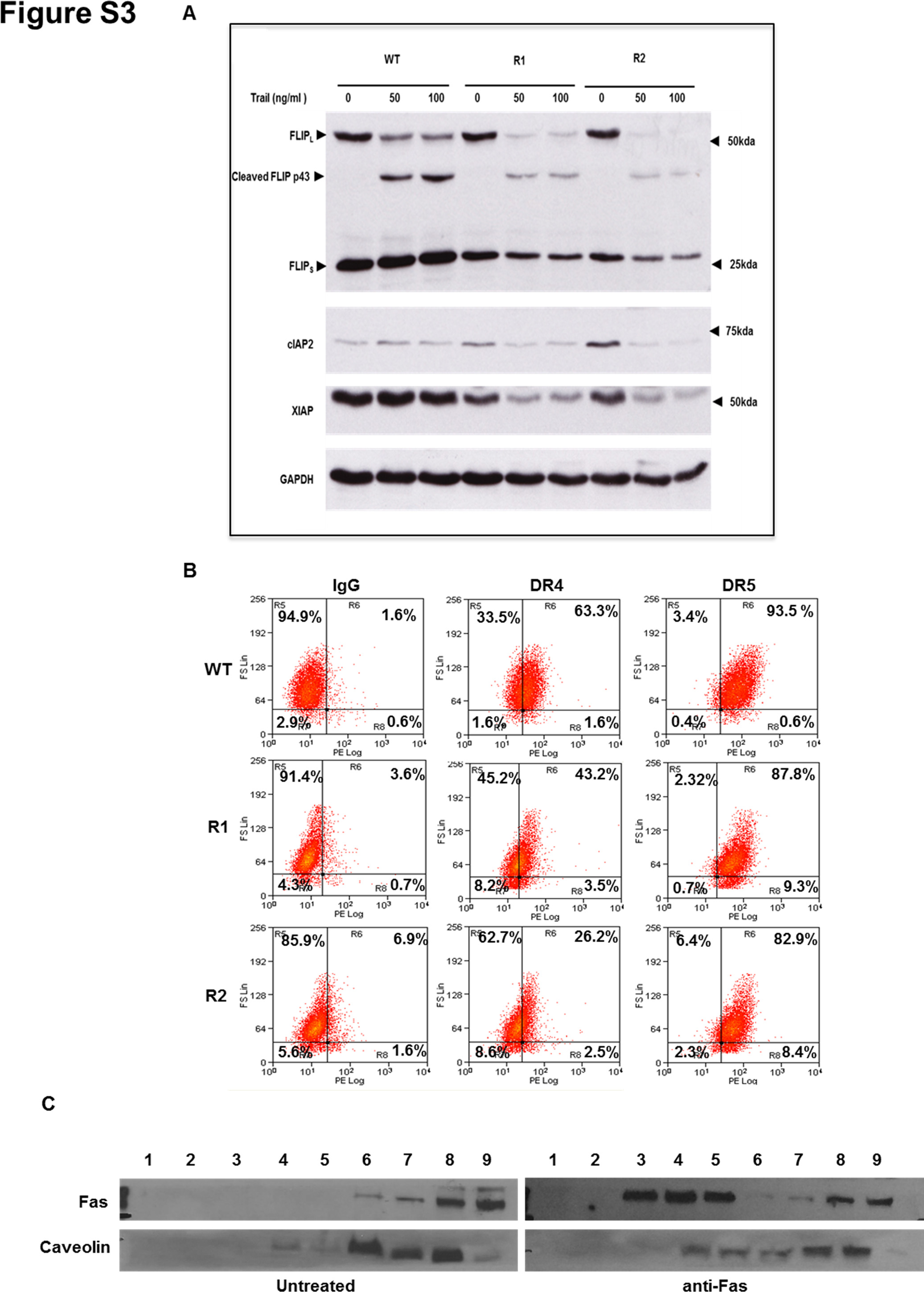

Supplement: Supplementary Figure S3 [file cddis2015299x4.tif]

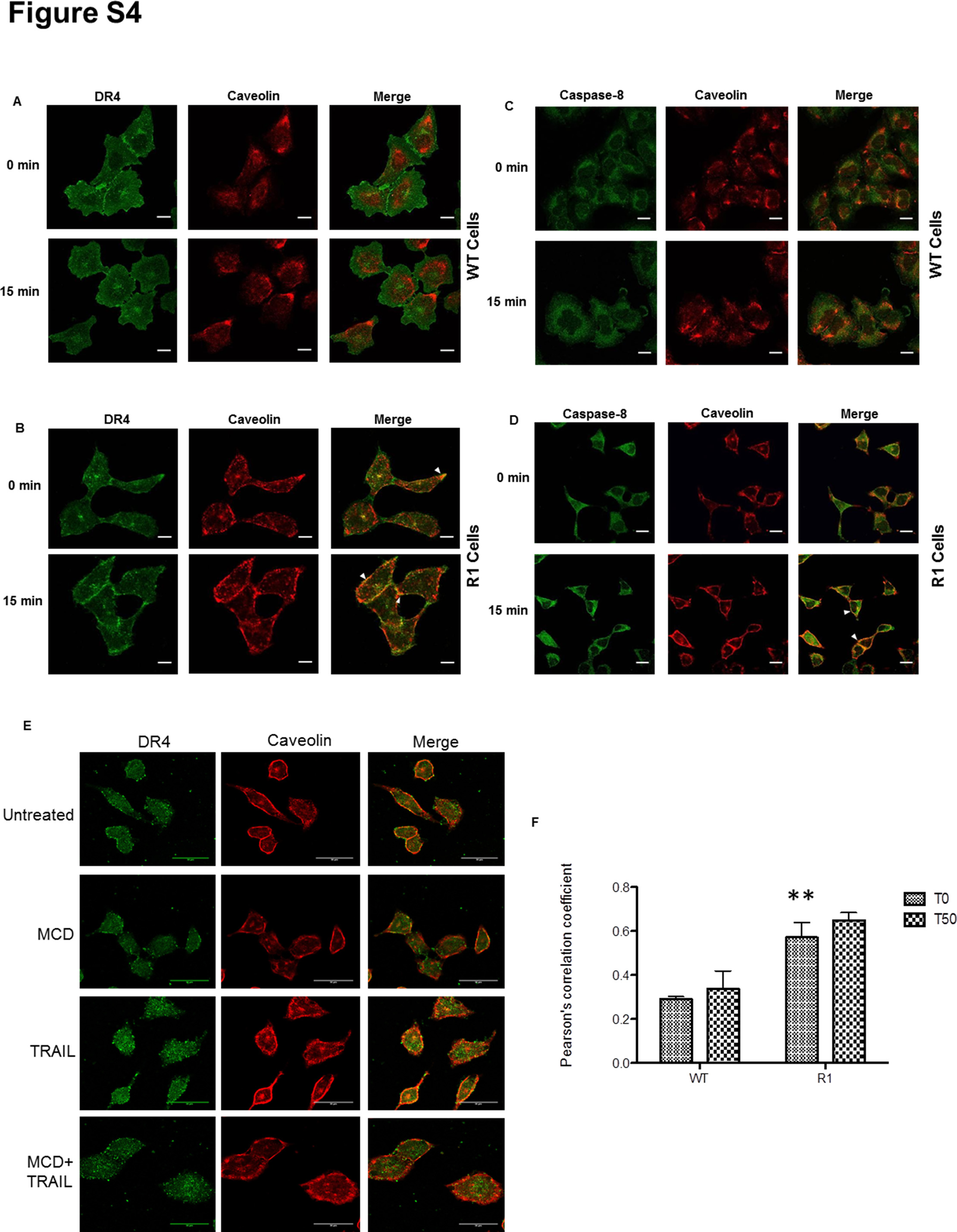

Supplement: Supplementary Figure S4 [file cddis2015299x5.tif]

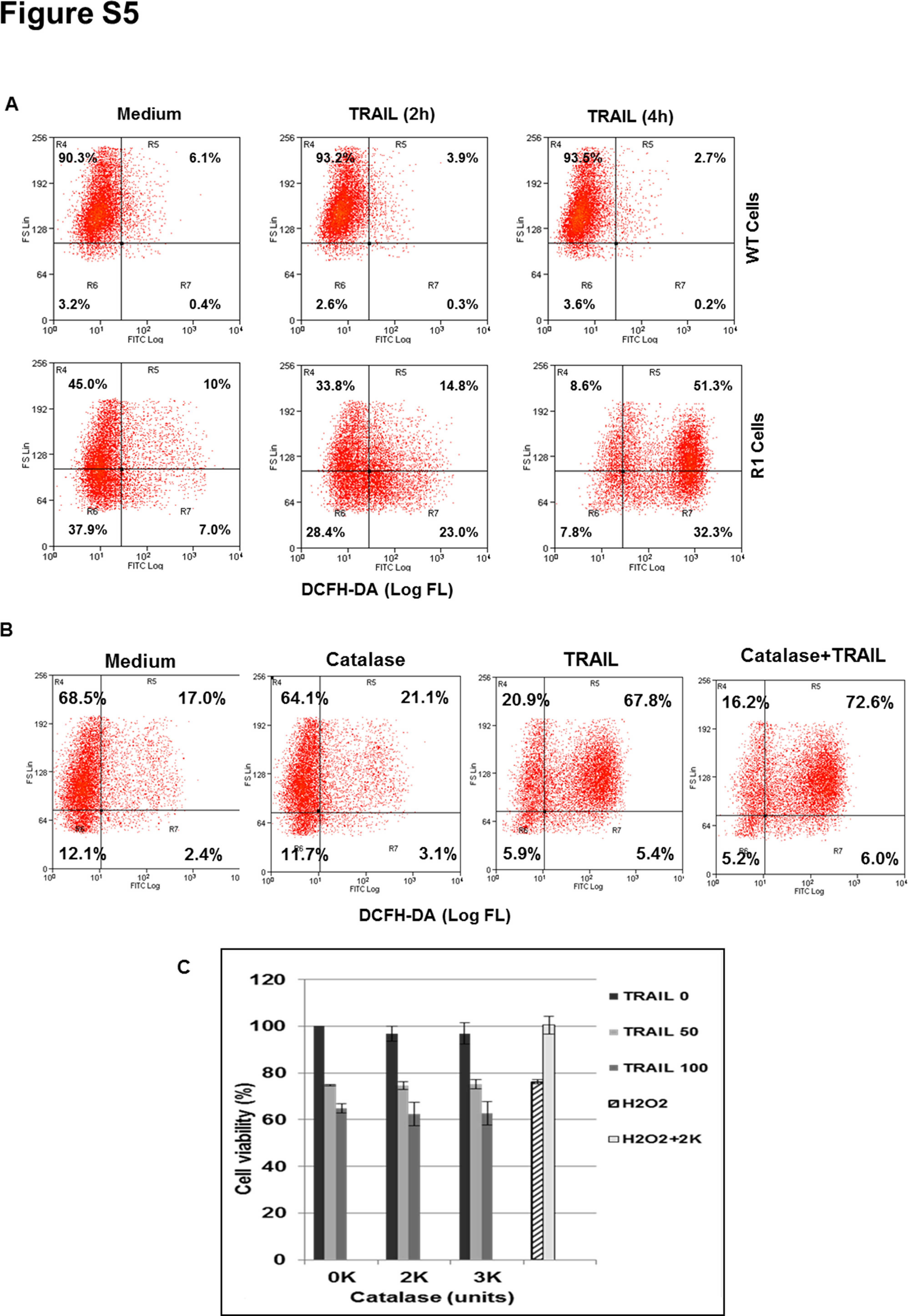

Supplement: Supplementary Figure S5 [file cddis2015299x6.tif]

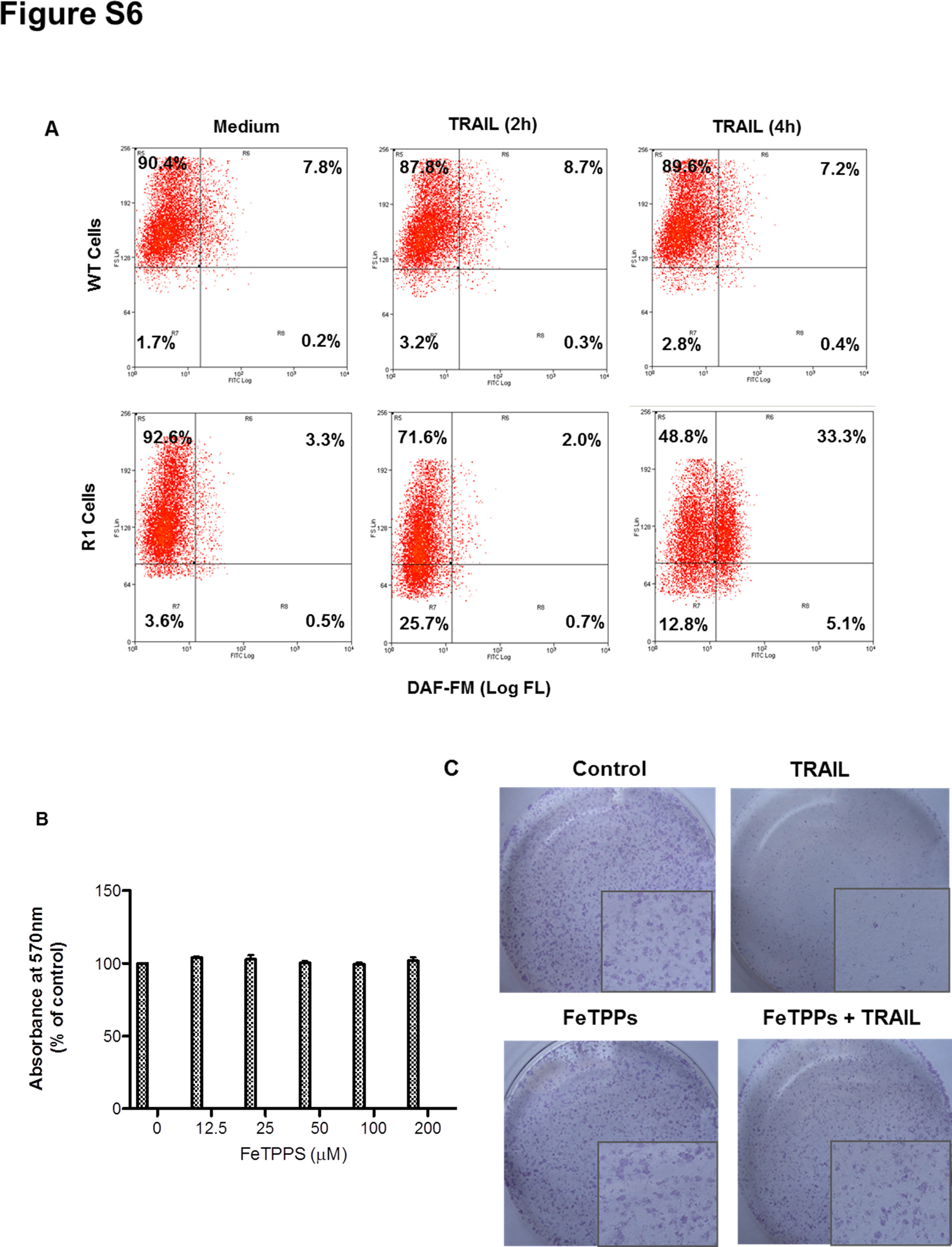

Supplement: Supplementary Figure S6 [file cddis2015299x7.tif]

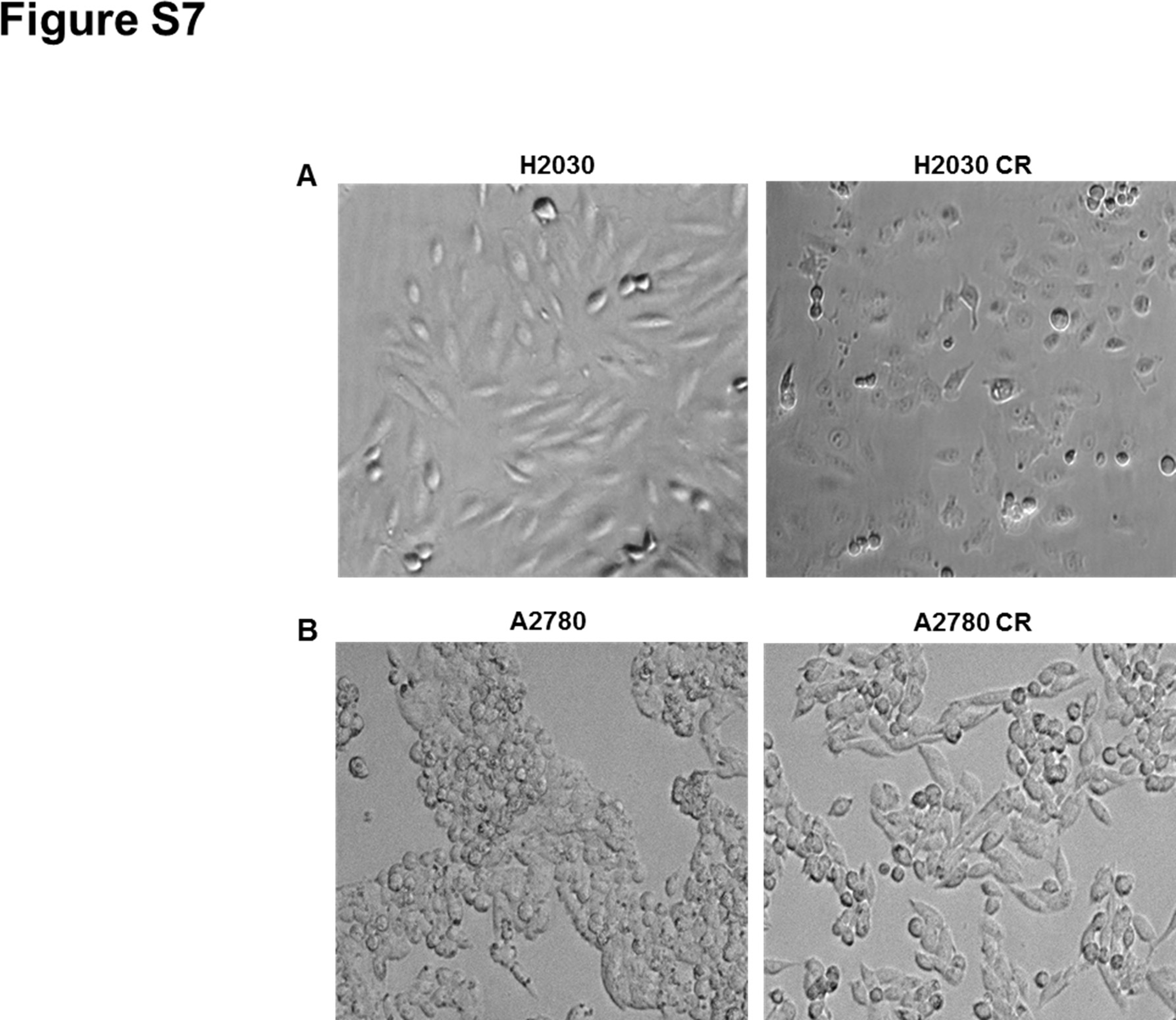

Supplement: Supplementary Figure S7 [file cddis2015299x8.tif]

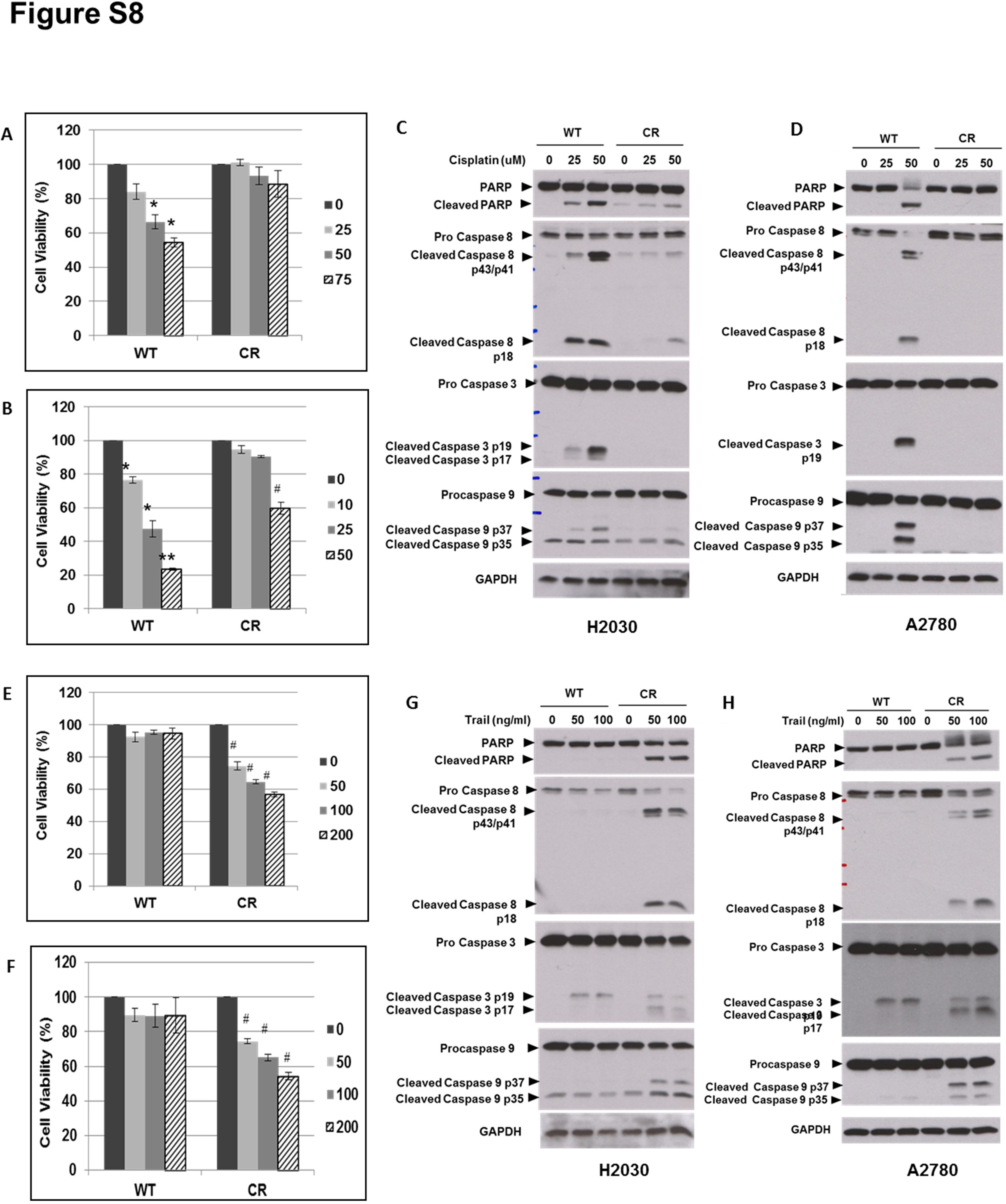

Supplement: Supplementary Figure S8 [file cddis2015299x9.tif]
