# Supplementary material for: Activation of surrogate death receptor signaling triggers peroxynitrite-dependent execution of cisplatin-resistant cancer cells
Source: Cell Death Dis. 2015 Oct 22;6(10):e1926–. doi: 10.1038/cddis.2015.299 (PMC4632318; doi:10.1038/cddis.2015.299)
Supplement: Supplementary Figure Legends [file cddis2015299x10.docx]

**Supplementary Figure S1: (A)** Representative photomicrographs showing the general cellular morphology of WT A549 cells and the cisplatin resistant clones R1 and R2. (**B**) Glucose deprivation reduces cell viability in cisplatin-resistant R1 cells. WT and R1 cells were treated with 2-deoxyglucose (mM) at the indicated doses for 24 h and cell viability was determined by MTT assay and expressed as % of untreated control. Data shown are Mean±SD of at least 3 independent experiments. *p < 0.05 **p < 0.005 compared to WT at the respective doses. (**C**) Overexpression of Bcl-2 does not confer protection against TRAIL-induced apoptosis in cisplatin-resistant R1 and R2 cells. R1 or R2 cells were transiently transfected with 1μg of pcDNA or pcDNA/Bcl-2 plasmid for 24 h. Cells were then treated with 50ng/ml of TRAIL for 24 h. Cell viability was determined by MTT assay and expressed as % of untreated control. All data represent mean ±SD of at least 3 independent experiments. Lower panels show Western blot analysis of Bcl-2 overexpression in R1 and R2 cells following 24 h post-transfection. Beta actin was used as loading control. (**D**) Cisplatin abolishes clonogenic abilities of parental WT but not the cisplatin-resistant cells. A549 WT , R1 and R2 cells were treated with the indicated doses of cisplatin for 24 h before being re-seeded onto 100mm petri dishes and allowed to form colonies for over 10 days. Following which, colonies formed were stained with crystal violet for assessment. Data shown are representative of at least 3 independent experiments. (**E**) Western Blot analysis of Apaf-1, caspase-8 and Bak protein expression from whole cell lysates of WT, R1 and R2 cells. GAPDH was used as loading control.

**Supplementary Figure S2: TRAIL mediates processing and activation of pro-caspases in cisplatin-resistant cells. (A)** WT and cisplatin-resistant cells were treated with the indicated doses of TRAIL for 24 h. Whole cell lysates were assayed for caspase-3 and 8 processing as well as PARP cleavage by Western blot analysis. **(B)** A549 WT, R1 and R2 cells were treated with the indicated doses of TRAIL (ng/ml) for 4 h, followed by staining with FITC-labeled annexin V and analyzed by flow cytometry. Data shown are representative of 3 independent experiments. (**C**) WT and R1 cells were exposed to 50ng/ml of TRAIL for varying durations as indicated. Total cell lysates were used to assess caspase-3, -6, -8, and -9 activation levels using fluorescence-conjugated substrates. Caspase activity was normalized to total protein and expressed as fold increase over untreated cells from each time-point. Data are Mean±SD of 3 independent experiments. * p< 0.05, ** p < 0.01, and *** p < 0.005 compared to WT cells at the respective doses*.* **(D)** R1 cells were pretreated with the indicated doses of caspase 8 inhibitor, Z-IETD for 1 h followed by TRAIL (50ng/ml) for 24hr. Cell viability was assessed by MTT assay and expressed as % of untreated cells. Data are expressed as Mean±SD of at least three independent experiments.

**Supplementary Figure S3: TRAIL significantly downregulates anti-apoptotic proteins in R1 and R2 cells. (A)** A549 WT, R1 and R2 cells were treated with the indicated doses of TRAIL for 24 h. Whole cell lysates were then collected to assay for cFLIP, cIAP2 and XIAP by Western blot analysis. GAPDH was used as loading control. **(B)** Surface expression of TRAIL receptors DR4 and DR5 in cisplatin-resistant cells. A549 WT, R1 and R2 cells were analyzed for the basal surface expression of DR4 and DR5 by staining with PE-conjugated mouse monoclonal anti-human DR4 or DR5 and sorted through flow cytometry. Non-specific IgG2B was used as an isotype control. A density plot; y-axis: forward scatter, x-axis: PE fluorescence (log). **(C)** Redistribution of the FAS receptors into the lipid rafts in the cisplatin-resistant R1 cells. Following Fas activating Ab (0.25μg/ml) treatment for 15 min, R1 cells were subjected to discontinuous sucrose density gradients of Triton X-100 cell lysates for separation of lipid raft and nonraft fractions. Fractions 1-9 were examined by Western blots for the presence of FAS receptors. Lipid raft fractions 5 and 6 were identified by Western blots by using lipid raft marker caveolin-1.

**Supplementary Figure S4: Co-localization of DR4 and DISC components with lipid rafts in the R1 cells. (A**) WT and **(B**) R1 cells were treated with 50ng/ml of TRAIL, fixed, permeabilized and stained with anti-DR4 (green) or anti-caveolin-1 (red) and analyzed by confocal microscopy. **(C**) WT and **(D**) R1 cells were treated with 50ng/ml of TRAIL, fixed, permeabilized and stained with anti-caspase-8 (green) or anti-caveolin-1 (red) and analyzed by confocal microscopy. Scale bars: 10 pixels (**E)** R1 cells were pretreated with MCD (4mM) for 1 h followed by 50ng/ml of TRAIL. The cells were then fixed and stained with anti-DR4 (green) or anti-caveolin-1 (red) and analyzed by confocal microscopy. Scale bars: 30μm **(F)** Quantitation of co-localization of DR4 and caveolin was carried out with Pearson’s correlation coefficient analysis. Arrows indicate co-localization.

**Supplementary Figure S5: TRAIL induces intracellular ROS/RNS accumulation in cisplatin-resistant R1 cells. (A)** WT and R1 cells were treated with 50ng/ml of TRAIL for 2 and 4 h. Cells were analyzed by flow cytometry for ROS production using redox sensitive probe, DCFH-DA. (**B**) R1 cells were preincubated for 1 h with 2000 units of catalase followed by 50ng/ml of TRAIL for 4 h and ROS was measured as in A. (**C**) R1 cells were pre-incubated with the indicated doses (k=1000 units) of catalase for 1 h followed by 50 or 100ng/ml of TRAIL for 24 h. Exogenous H_2_O_2_ (400μM) was used as a positive control. Cell viability was assessed by MTT assay and expressed as % of untreated cells. Data are expressed as Mean±SD of at least three independent experiments.

**Supplementary Figure S6: TRAIL induces an increase in intracellular NO in R1 cells and FeTPPS can rescue TRAIL-mediated cell death. (A)** R1 cells were treated with 50ng/ml of TRAIL for 4 h. Cells were subsequently harvested and analyzed by flow cytometry for ROS production using NO specific probe DAF-AM. At least 10,000 events were analyzed. Data are expressed as Mean±SD of at least three independent experiments. **(B)** FeTPPS does not interfere with the MTT assay. R1 cells were treated with increasing doses of FeTPPS for 24 h. Cell viability was assessed by MTT assay and expressed as % of untreated cells. Data are expressed as Mean±SD of at least three independent experiments. **(C)** FeTPPS restored long-term colony forming ability of R1 cells following TRAIL treatment. Cells were preincubated with 50μM FeTPPS for 1h followed by TRAIL treatment for 4h before being re-seeded onto 100mm Petri dishes and allowed to form colonies for over 10 days. Following which, colonies formed were stained with crystal violet for assessment. Data shown are representative of at least 3 independent experiments

**Supplementary Figure S7:** Representative photomicrographs showing the general cellular morphology of the **(A)** H2030 and cisplatin-resistant H2030 CR, **(B)** ovarian A2780 WT and cisplatin-resistant A2780 CR cells (Magnification: 40X).

**Supplementary Figure S8: Cisplatin resistant variants of NSCLC H2030 and ovarian cancer A2780 cells also exhibit increased TRAIL sensitivity. (A**) H2030 (**B**) A2780 WT and CR cells were treated with varying doses of cisplatin (μM) for 24 h and cell viability was determined by MTT assay and expressed as % of untreated control cells. Western blot analysis of caspase-3 and caspase-8 processing as well as PARP cleavage following 24 h of cisplatin (μM) treatment in (**C**) H2030 (**D**) A2780 WT and CR cells. GAPDH was used as loading control. (**E)** H2030 and (**F**) A2780 WT and CR cells were treated with varying doses of TRAIL (ng/ml) for 24 h and cell viability was determined by MTT assay and expressed as % of untreated control. Western blot analysis of caspase-3 and caspase-8 processing as well as PARP cleavage following 24 h of TRAIL (ng/ml) treatement in (**G**) H2030, (**H)** A2780 WT and CR cells. GAPDH was used as loading control. Data shown are Mean±S.D. of at least three independent experiments.
